# Design of an Ultra-Sensitive Multi-Resonant Moore Fractal SRR Microwave Sensor for Non-Invasive Blood Glucose Monitoring

**DOI:** 10.3390/s26082306

**Published:** 2026-04-09

**Authors:** Zaid A. Abdul Hassain, Malik J. Farhan, Taha A. Elwi

**Affiliations:** 1 Electrical Engineering Department, Mustansiriyah University, Baghdad 1004, Iraq; zaidasaad_79@uomustansiriyah.edu.iq (Z.A.A.H.); malik.jasim@uomustansiriyah.edu.iq (M.J.F.); 2 Department of Automation and Artificial Intelligence Engineering, College of Information Engineering, Al-Nahrain University, Baghdad 10070, Iraq

**Keywords:** fractal resonator, SRR, microwave biosensor, non-invasive glucose monitoring, S-parameters, physics-informed deep learning, electromagnetic characterization, biomedical sensing

## Abstract

This study details the design and development of an ultra-sensitive microwave sensor for non-invasive blood glucose monitoring, achieved by analyzing variations in the response of a split-ring resonator (SRR) through advanced engineering methodologies. There were three design phases in the development process. In the first phase, a standard SRR design was used. It had a resonant frequency of 2.975 GHz in S_21_ and a sensitivity of only 0.0032 dB/(mg/dL). In the second phase, an interdigital capacitor (IDC) was added to the SRR structure. This made it work better and made it more sensitive, with a sensitivity of 0.015 dB/(mg/dL) at 4.1 GHz. The third phase was to use a fourth-order Moore fractal geometry to improve the resonance properties of the design a lot. From the obtained S_11_, the maximum sensitivity was 0.042 dB/(mg/dL), which was a huge improvement in sensing efficiency compared to earlier designs. Several resonant frequencies were recorded between 4.84 and 7.56 GHz. The addition of the fractal structure made the electromagnetic field stronger in the resonant space and made the waves interact more with small changes in the biological medium, all without changing the sensor’s size (80 mm × 40 mm). These results show that fractal architecture is a promising way to create non-invasive, accurate, and easily integrated sensors in biological systems that can continuously measure blood glucose levels.

## 1. Introduction

**Figure 1:**
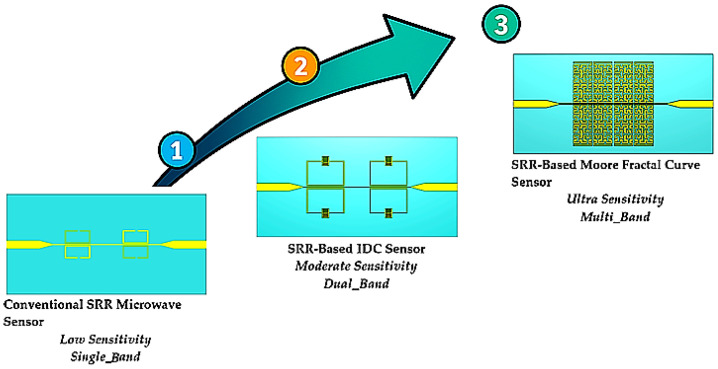
Activity procedures performed to design ultra-sensitive microwave sensors for glucose monitoring. The numbers (1–3) indicate the progressive design stages: (1) conventional SRR sensor, (2) SRR with interdigital capacitor (IDC), and (3) Moore fractal SRR sensor. The arrow represents the evolution of the design toward higher sensitivity, while the color gradient illustrates the improvement in performance from low to ultra-high sensitivity.

Diabetes is a chronic condition that requires early detection and treatment to reduce risks and enhance patient health. Over the last hundred years, the rise in diabetes cases has mostly been blamed on poor lifestyle choices, like not working out and eating an unbalanced diet. According to the World Health Organization (WHO), the number of adults with diabetes has gone up from 4.7% in 1980 to 8.5% in 2014. Diabetes was directly responsible for about 1.6 million deaths in 2015 alone [1,2]. To avoid problems that come with diabetes, such as heart disease, kidney damage, vision loss, a higher risk of stroke, and damage to the lower limbs, it is important to have a complete diabetes care plan. People with diabetes need to check their blood sugar levels every day. You usually have to take many measurements throughout the day, and each test needs a lancet. Most glucose meters use electrochemical processes, which means you have to put a drop of blood on a test strip. Regular intrusive monitoring can be detrimental to your health, like when it contaminates the testing equipment [3,4]. A non-invasive approach could mitigate the prevalent risks, inconveniences, and irritations linked to conventional testing. Researchers have been studying different ways to monitor blood sugar levels without cutting into the skin for more than 40 years [5]. For instance, studies in [6,7,8] have explored the analysis of urine and tears as potential non-invasive methods for glucose monitoring. Even though these methods let you check your blood sugar levels without having to do anything invasive, blood has a lot more glucose than saliva, sweat, and tears. Also, these methods are not the best for long-term observation. However, making a good non-invasive glucose sensor is hard because it needs to be very accurate, cheap, easy to use, small, and able to handle changes in temperature and body fluids. For people with diabetes, the best way to continuously monitor their blood sugar levels is to do so without touching them. They need to do these activities often, like before meals, two hours after meals, before bed, and so on [2]. In this context, microsensors are becoming more popular because they can continuously and non-invasively monitor glucose levels without getting in the way of the patient’s daily routine. For instance, we can use the differences in dielectric properties in [6] to monitor glucose levels without physical contact with the patient. This is what a radio wave transmission-based glucose meter that works at frequencies between 5 GHz and 12 GHz does. The main problem with this technology is that it needs to use high frequencies to reduce the effect of skin on measurement accuracy. The “photoplethysmography” method, which is a concept in [9], is different. But this kind of sensor has the problem of needing another sensor to measure the heart rate [10]. In the last twenty years, there has been more and more interest in wireless technologies and their many uses, such as using microwave or radio frequency (RF) technologies to measure blood glucose levels. Moreover, the aspiration to develop medical diagnostic devices originates from the examination of the interaction between electromagnetic waves and physiological tissues, encompassing the evaluation of the dielectric properties and anomalies of tissues [11,12,13,14,15]. One of the most important things to think about when designing a microwave or radio frequency structure is the properties of the dielectric material. Their importance is because these properties control how electromagnetic waves move through the structure. Advancements in body-centric and portable wireless communication systems, along with the potential for implanted sensors to monitor biological processes, have significantly augmented research into the effects of electromagnetic waves on the human body. One study examined the utilization of a dielectric resonator operating at 1.68 GHz to detect alterations in glucose levels by monitoring variations in resonance frequencies. However, the detection accuracy of this type of sensor was considerably below the 5 mg/mL threshold required for human measurements. There have been setups for resonant cavities made before [16,17], and they are meant to work at frequencies between 2 and 3 GHz. Some studies have used antenna sensors with frequency ranges of 1 to 2.5 GHz [18] and 5 to 8.5 GHz [19]. Moreover, open-ended waveguide structures have been employed to investigate the influence of fluctuating glucose concentrations on the dispersion coefficients (S-parameters) of waveguides up to 20 GHz [20,21]. In [22], a metamaterial-based microfluidic sensor was suggested for measuring the amount of glucose in water. The proposed sensor employs a capacitor positioned between the fingers within the resonator, facilitating high sensitivity for the assessment of insulating liquids. A patch antenna that works at 2.4 GHz and 5.8 GHz is designed, with deionized water and glucose inside for medical use, and the antenna matching changes based on glucose levels. In [23], the creation of patch antenna components with phantoms for an antenna that works at 4.75 GHz is described. The research encompassed the assessment of various liquid phantoms, including porcine blood and physiological solutions, with glucose concentrations between 150 and 550 mg/dL. The results showed a linear frequency change at 5 MHz, and there was no link between the readings and changes in temperature or sample volume. In [24], a specific optimization process is used to make microwave sensors that respond the same way no matter what tissues other than blood are present. To assess the proposed concept and design strategy, various sensor configurations along with their corresponding resolution metrics are considered [25]. To differentiate concentrations within the range of [100–300] mg/dL, numerical results confirm the feasibility of attaining an optimal balance between sensitivity to blood glucose levels and measurement stability against unwanted phantom variations. In [26], a dual-frequency microwave split-ring resonator is shown for liquid samples. In [27], a similar method is suggested, where two-resonant frequency dual-sensing is used. A non-invasive sensor for measuring glucose levels is shown. Microwave resonators come in many shapes, as seen in [4,28,29,30]. You can find more intriguing designs with high Q-factors that use split-ring resonators in [31,32]. This study proposes a non-invasive microwave sensor for monitoring blood glucose levels, utilizing a split-ring resonator (SRR) structure. Two important development strategies were used to improve the sensor’s sensitivity and overall performance based on the original SRR design. The first improvement was adding IDC structures to the layout of the resonator. The goal of this change was to make the local electric field stronger in the sensing area, which would improve the interaction between the electromagnetic waves and the biological tissue that contains glucose. As a result, the sensor became more sensitive due to the tighter confinement of the electric field, which allowed it to react more strongly to changes in permittivity. The second step in the development process added a Moore fractal geometry to the SRR structure. Fractal-based geometries are known to enable multiband behavior, compact dimensions, and extended surface current path lengths. Using a fourth-order Moore fractal configuration greatly increased the effective interaction area and electric field distribution. These improvements made the sensor very sensitive to changes in the dielectric caused by glucose. This method made it possible to create a very sensitive microwave sensor that does not need to touch anything and can accurately detect even the smallest changes in glucose concentration, as shown in Figure 1.

This paper aims to have a high-sensitivity and non-invasive nature. The proposed sensor shows strong potential for integration into wearable biomedical devices or continuous glucose monitoring (CGM) systems, offering a promising alternative to traditional invasive blood glucose measurement techniques.

## 2. Conventional SRR Microwave Glucose Biosensor

### 2.1. Proposed Model

**Figure 2:**
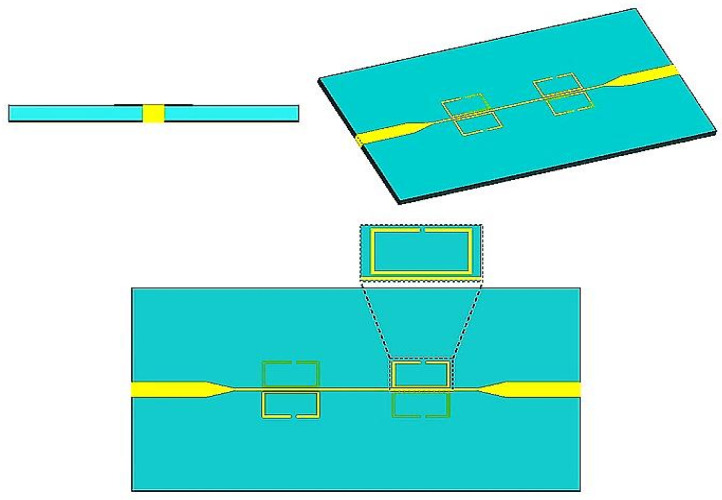
Schematic representation of the proposed sensor-based SRR.

The microwave characterization process uses the strong interaction between the sensor’s electric fields and the electro-dielectric properties of the biological material to focus on the fingertip tissue, which has blood that carries glucose. One of the best things about SRR structures is that they make the electric field in the capacitive gap very strong. Putting the fingertip tissue, especially the blood-rich area, in this zone makes the sensor very sensitive to small changes in permittivity caused by different glucose levels. This enables precise detection of dielectric variations via capacitive coupling [26,33,34]. The gap between the SRR structures has a very strong electric field. This procedure lets the inductive sensing method figure out the relative permittivity. Figure 2 shows that the proposed microwave sensor has four SRR configurations, with two split-ring resonators placed symmetrically at opposite ends of a microstrip transmission line. The figure also shows the geometric properties of the design. An SRR is a small piece of metamaterial that is designed to respond strongly to electromagnetic waves at a specific frequency. The structure usually has one or more concentric metallic rings with splits (gaps) in them. These splits break the path of the current and let electric fields form in small areas. When the SRR is shaped like a single-loop rectangle, it works like a sub-wavelength resonator. Because of its shape, it has inductance (L_r_) and capacitance (C_r_), which can be modeled as an LC tank circuit. The loop’s perimeter is what mostly determines the inductance. You can use a modified version of Grover’s equation for a rectangular current loop to get an idea of what it is.

(1) $$L_{r}=\mu_{o}P\left[ln\left(\frac{2P}{w+g}\right)-0.774\right]$$

(2) $$C_{r}=\epsilon \frac{wh}{g}$$

where: $\mu_{o}=4\pi \times \frac{10^{-7}H}{m}$.

P is the perimeter of the rectangular loop.

Where *L_r_* denotes the equivalent inductance of the rectangular SRR loop and *Cr* represents the equivalent capacitance associated with the SRR split gap. The parameter *μ*_0_ = 4*π* × 10^−7^ H/m is the permeability of free space. The variable P corresponds to the perimeter of the rectangular resonator, defining the total electrical length of the current path. The parameters www and g indicate the width of the metallic strip and the SRR split gap, respectively.

### 2.2. Theoretical Analysis

**Figure 3:**
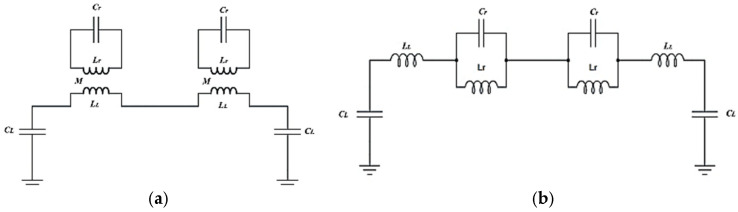
(**a**) The suggested sensor’s equivalent circuit model, (**b**) transformed equivalent circuit model.

Figure 2 shows that the SRR structures in the proposed sensor are magnetically connected to the microstrip line. Figure 3 shows the equivalent circuit model of the proposed sensor, which does not take into account the losses in the design. An inductor (Lr) and a capacitor (Cr) make up each SRR unit in the proposed equivalent circuit model. The microstrip line connects to these LC tank circuits through mutual coupling M. The proposed sensor design utilizes an inductor (L_L_) and a capacitor (C_L_) to emulate the microstrip line referenced in [35,36]:

(3) $$L_{L}=\left\{\begin{array}{c} \frac{60l}{c}ln\left[\frac{8h}{wf}+\frac{w}{4h}\right]\;\;\;\;\;\;\;\;\;\;\;\;\;\;\;\;\;\;\;\;\;\;\;\;\;\;\;\;\;\;\;\;\;\;\;\;\;\;\;\;\;\;\;\;\;\;\;\;\;\;\;\;\;\;\;\;\;\;\;\;\;\;\;\;\;\;\;\;\;for\;\frac{wf}{h}\leq 1\\ \frac{120\pi l}{c}\left[\frac{1}{\frac{wf}{h}+1.393+0.667\ln\left(\frac{wf}{h}+1.444\right)}\right]\;\;\;\;\;for\;\frac{wf}{h}>1 \end{array}\right.$$

(4) $$C_{L}=\left\{\begin{array}{c} \frac{\varepsilon_{r}l}{60cln\left[\frac{8h}{wf}+\frac{wf}{4h}\right]}\;\;\;\;\;\;\;\;\;\;\;\;\;\;\;\;\;\;\;\;\;\;\;\;\;\;\;\;\;\;\;\;\;\;\;\;\;\;\;\;\;\;\;\;\;\;\;\;\;\;\;\;\;\;for\;\;\frac{wf}{h}\leq 1\\ \frac{\varepsilon_{r}l\left[\frac{wf}{h}+1.393+0.667\ln\left(\frac{wf}{h}+1.444\right)\right]\;\;\;\;\;for\frac{wf}{h}>1}{120\pi c} \end{array}\right.$$

where L and CL represent the equivalent inductance and capacitance per unit length of the transmission line, respectively. The parameter εr denotes the relative permittivity of the substrate, w_f_ is the conductor width, and h is the substrate thickness.

**Figure 4:**
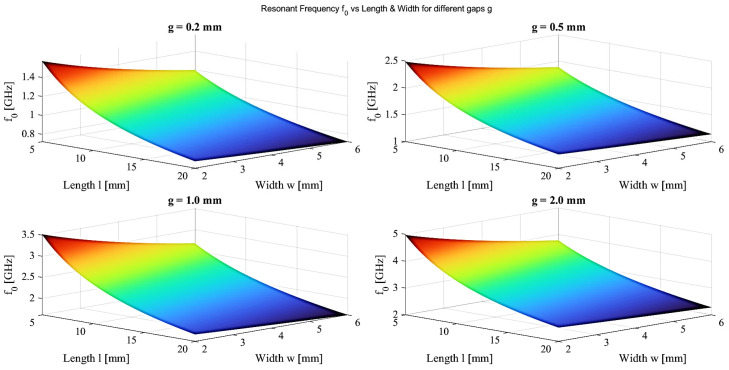
Variation of the resonance frequency f_0_ as a function of SRR length and width for different splits.

The circuit shown in Figure 3b is a modification of the corresponding circuit in Figure 3a [10]. Equation (3) states that the values of the tank’s capacitance and inductance determine the resonant frequency. Consequently, as illustrated in Figure 4, a number of relationships were created to compute the resonant frequency using the real sensor dimensions, which are represented by the length (L), width (w), and gap width (g), to determine the frequency of the proposed sensor. The following sensor dimensions were selected based on Figure 4. The optimized geometrical parameters of the proposed SRR sensor are summarized in Table 1.

**Figure 5:**
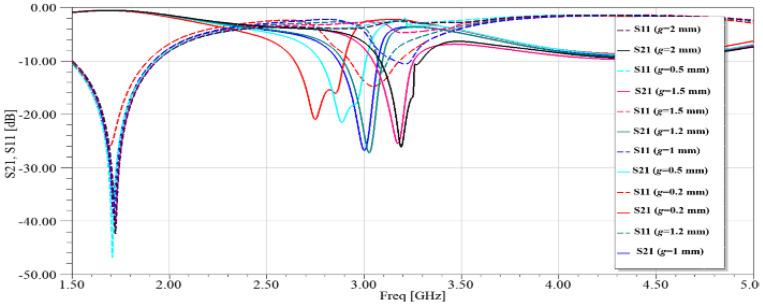
Simulation of SRR unit cell: (**a**) magnitudes of S-parameters (S_11_, S_21_).

**Figure 6:**
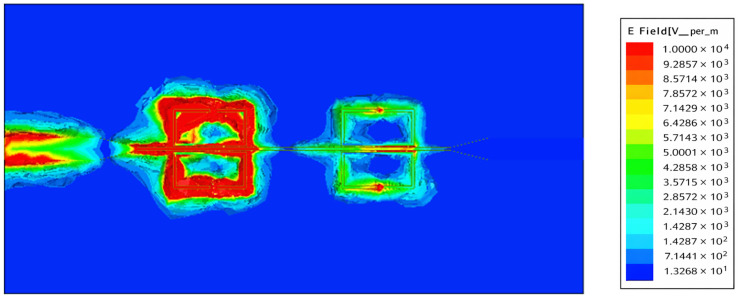
Electric field variation at frequency *f_o_* = 3 GHz.

**Figure 7:**
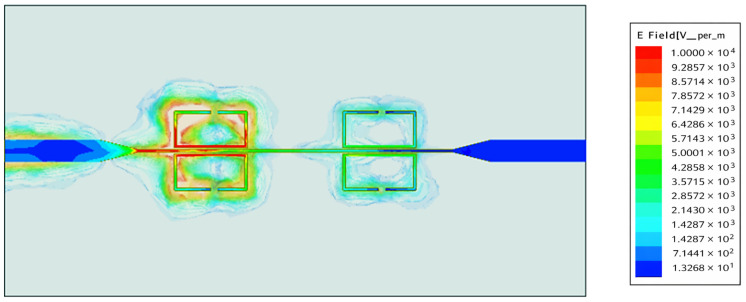
Current distribution at frequency *f_o_* = 3 GHz.

The gap width, g, is the main thing that affects the tank’s capacitance and, as a result, the strength of the electric field. Because of this, Figure 5 shows the evaluated S_21_ and S_11_ coefficients for different gap widths. Figure 6 shows how the electric field in the proposed sensor’s SRRs changes at a certain frequency (3 GHz). Figure 6 shows that the SRR connected to the microstrip line has almost no electric field changes in the middle of the edge, which is farthest away from the microstrip line. The electric field changes the most in the SRR gap, which makes it easy to test the dielectric properties of MUT tissue. At its resonance frequency, an SRR acts as a band-reject filter, stopping a signal from getting through at that frequency. This scenario happens when a microstrip transmission line inductively supplies a single loop of the SRR, as shown in Figure 7.

**Figure 8:**
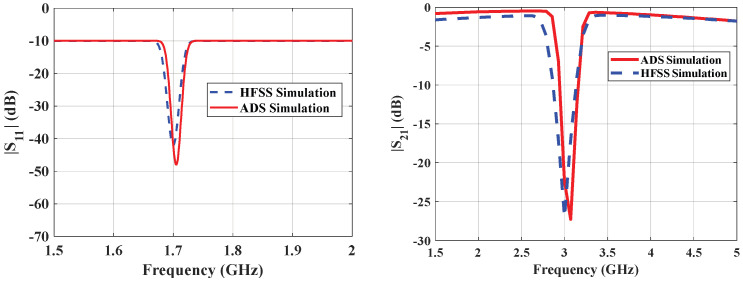
Comparison of EM simulation and circuit theory-based transmission of SRR properties.

Using the ADS, the values of the lumped elements in Figure 3b and the transformed equivalent circuit diagram are retrieved and tabulated in Table 2. Figure 8 compares the S_21_ and S_11_ plots with the ADS circuit.

### 2.3. Quantitative Evaluation

**Figure 9:**
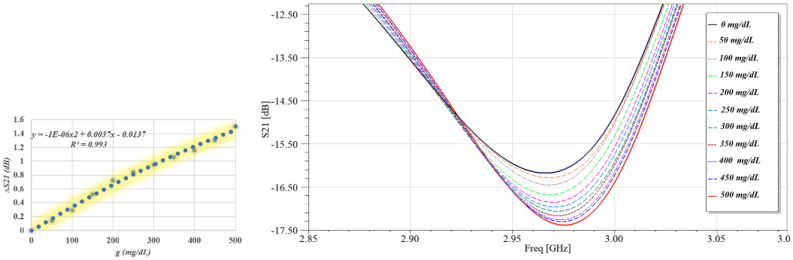
Variation of the transmission coefficient ∣S_21_∣ around the resonance frequency.

**Figure 10:**
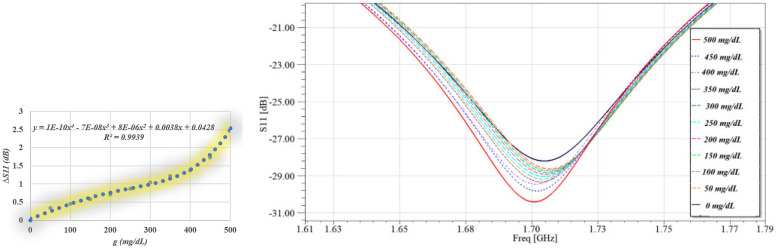
Variation of the reflection coefficient ∣S_11_∣ around the resonance frequency.

This section examines the performance of the suggested sensor when it is used to monitor blood sugar levels without making skin incisions using the High-Frequency Structure Simulator (HFSS). The distance between the MUT (finger phantom) and the sensor surface is z = 1 mm. The glucose samples are changed from 0 mg/dL to 500 mg/dL using the parametric sweep function. This enables us to observe how variations in glucose levels impact the interrogating antenna’s electric field and scattering response. The sensor’s S_11_ and S_21_ responses to eleven distinct glucose concentrations, ranging from 0 mg/dL to 500 mg/dL, are depicted in Figure 9 and Figure 10. The resonance depth in these sensing modes varies with variations in glucose levels. The average sensitivity at each S_11_ and S_21_ resonance is displayed in dB/mg/dL in Table 3. Table 3 summarizes the average sensitivity of the proposed sensor at its dominant resonance frequencies, extracted from both the transmission (S_21_) and reflection (S_11_) responses. The sensor exhibits a resonance at 2.975 GHz in the transmission mode, achieving an average sensitivity of 0.0032 dB/(mg/dL), while a higher sensitivity of 0.0052 dB/(mg/dL) is observed at 1.7 GHz in the reflection mode. This comparison highlights the superior sensitivity of the S_11_-based sensing mechanism, indicating a stronger interaction between the localized electromagnetic fields and glucose-induced dielectric variations at the reflection resonance.

## 3. SRR-Based IDC Sensor

Initially, the design was improved by incorporating IDC elements into the resonator structure. This enhancement was intended to amplify the localized electric field within the sensing area, thereby strengthening the interaction between the microwave signal and the glucose-rich biological tissue.

### 3.1. Sensor Configuration and Features

The resonant frequency of an SRR depends solely on the resonator’s effective capacitance. Therefore, improving sensor performance focuses on enhancing this capacitance. To increase the sensing area and response sensitivity, an interferometric capacitor (IDC) structure was introduced into the SRR design, aiming to increase the effective capacitance and thus improve the device’s response to small changes in the material under test. The strength of an SRR lies in its ability to confine the electric field within a very small gap. However, when an IDC structure is inserted into this gap, the electric field is distributed over a much wider area, allowing for increased interaction with the biomaterial (such as blood), thus improving the sensor’s accuracy and response to very small changes in glucose. The IDC architecture is widely used in microwave applications, especially in sensors, filters, resonators, and matching networks.

**Figure 11:**
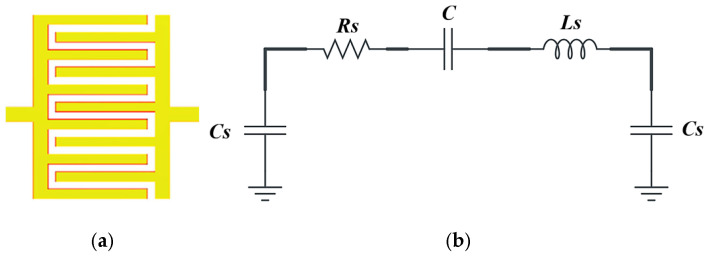
IDC: (**a**) EM structure; (**b**) equivalent circuit.

An interdigital capacitor is a structure consisting of an array of overlapping metal “fingers” on the surface of an insulating substrate, with each finger connected to the opposite potential of the adjacent finger, generating a concentrated electric field between each pair of fingers. The IDC can be represented by an equivalent circuit containing (as shown in Figure 11). The total capacitance due to interference between the pins (*C*). Parasitic inductance is due to current distribution around the metal terminals (*Ls*), the self-capacitance between each pin and the ground substrate (Cs), and the resistance loss due to current in the metal and the insulation losses (*Rs*) [37]. The total capacitance generated between the IDC fingers is given by the elliptic integral-based model as [38]:

(5) $$C=\varepsilon_{e}\varepsilon_{o}\frac{K\left(k\right)}{K\left(k^{\prime }\right)}2\left(N-1\right)L$$

where N is the number of fingers, $k=\frac{w}{w+2S}$, $k^{\prime }=\sqrt{1-k^{2}}$, $\varepsilon_{e}≅\frac{\varepsilon_{r}+1}{2}$, and $K\left(k\right)\;and\;K^{\prime }\left(k\right)$ are the complete elliptic integrals of the first kind and its complement given as:

(6) $$K\left(x\right)=\int_{0}^{\frac{\pi }{2}}\frac{1}{\sqrt{1-x^{2}{sin}^{2}\theta }}d\theta$$

$R_{s}=\frac{1}{\sigma \delta w}$, where δ is the skin effect and σ is the conductor conductivity.

(7) $$L_{s}=\mu_{o}\frac{L}{w}\left\{ln\left(\frac{2L}{w}\right)+0.5\right\}$$

(8) $$C_{s}=\varepsilon_{r}\varepsilon_{o}\frac{wL}{h}$$

The parameter Rs represents the surface resistance, where σ is the conductor conductivity in s/m and δ is the skin depth in m.

A microwave SRR sensor’s sensitivity is greatly increased when the IDC structure is incorporated into it since it raises the total effective capacitance, resulting in a greater concentration of electric fields between the fingers, a greater interaction surface area with the sample being tested (MUT), such as blood or tissue, and more responsiveness to slight variations in permittivity in the MUT [19]. When a material such as finger tissue enriched with blood (which has a variable glucose content) is placed on a surface, the change in the electrical permittivity of this material leads to a change in *ε_r_*, which in turn leads to a change in the capacitance, C, and thus to a change in the resonant frequency.

**Figure 12:**
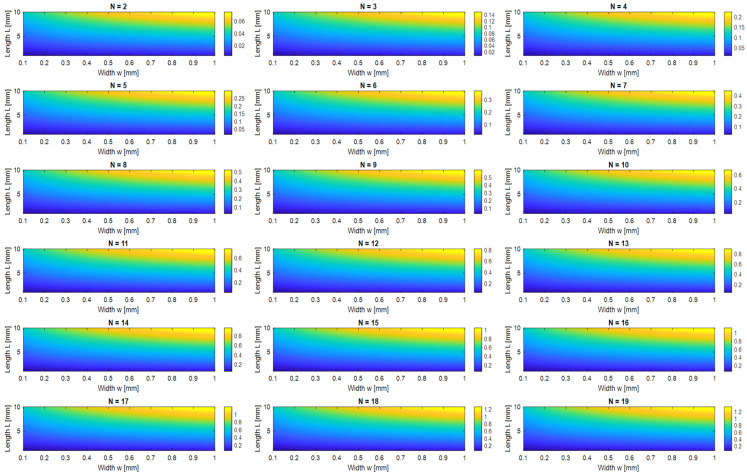
Parametric maps of IDC capacitance versus finger width www and length LLL for varying numbers of fingers; N = 2–19.

The capacitance behavior of the IDC structure was studied in this work in relation to the number of fingers (*N*), which ranged from 2 to 17, as well as the finger length (*L*) and width (*w*), as shown in Figure 12. An accurate analytical model incorporating elliptic integrals was used to estimate the capacitance values. These results clearly show that increased surface area and fringe electric field coupling cause the effective capacitance to rise proportionately with either finger length or width. Additionally, the overall capacitance increases almost linearly as the number of fingers N increases, providing a simple and scalable way to adjust the IDC-based sensor’s resonant response. The corresponding parasitic inductance L and series resistance *Rs* remained comparatively low and constant despite alterations in geometry, maintaining high-quality factor (Q) performance and reducing energy dissipation. These qualities are especially useful in sensing applications, where detectable shifts in the resonant frequency can be caused by even little modifications to the material under test’s (MUT) dielectric characteristics. The ability to finely control *C_eff_* without significantly altering *Leff* enables precise tuning of the resonance frequency.

### 3.2. Proposed SRR-Based IDC Sensor Analysis

**Figure 13:**
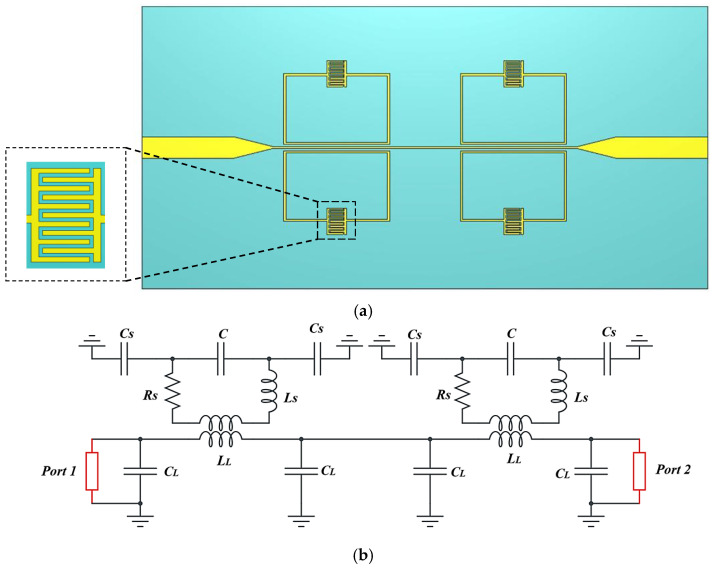
Proposed SRR-based IDC sensor: (**a**) EM structure, (**b**) equivalent circuit model.

Figure 12 shows the proposed SRR-based IDC sensor EM structure (Figure 1) and the equivalent circuit model (Figure 1). In general, the microstrip line can be modeled by the inductance *L_L_* and the capacitance *C_L_*, as shown in Figure 1. At the resonant frequency, the IDC-SRR can be represented by a series combination of inductance *L_S_*, capacitance C, and resistance *R_S_* of the IDC. The design of the proposed SRR-based sensor with an integrated IDC is governed by its equivalent Leff *C_eff_* resonance behavior. The resonant frequency is defined by:

(9) $$f_{o}=\frac{1}{2\pi \sqrt{L_{eff}C_{eff}}}$$

where *L_eff_* is the total inductance associated with the ring’s geometry and self-inductor IDC fingers. *C_eff_* is the capacitance, enhanced by the IDC structure. To determine the sensor dimensions, the outer length and width of the SRR are selected based on the desired frequency range. The inductance Leff increases with the total ring perimeter, while the capacitance *C_eff_* is primarily controlled by the IDC finger length, width, spacing, and number of fingers. By adjusting these physical parameters, namely, the IDC dimensions and ring size, the sensor’s resonant frequency can be accurately set within the desired GHz range. Moreover, larger IDC dimensions and more fingers lead to higher capacitance and, consequently, lower *f_o_*, offering enhanced sensitivity to changes in the dielectric properties of the sensing target, such as glucose-rich biological tissues. The following sensor dimensions (Table 4) were selected based on Figure 13.

**Figure 14:**
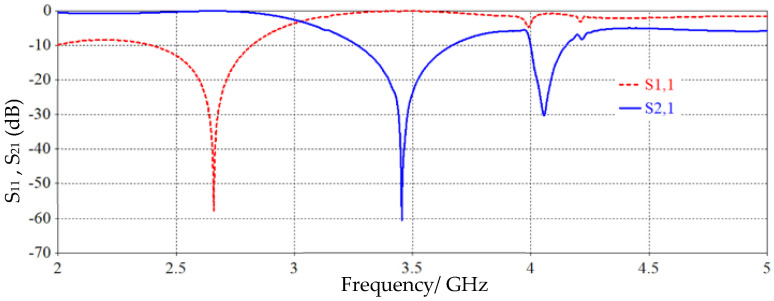
S_11_ and S_21_ responses of the proposed SRR-based IDC sensor.

Figure 14 shows the proposed SRR-based sensor S_11_ and S_21_ response. The sensor S_21_ response shows two resonance modes at 3.42 GHz and 4.15 GHz, and the S_11_ response shows resonance at 2.65 GHz.

**Figure 15:**
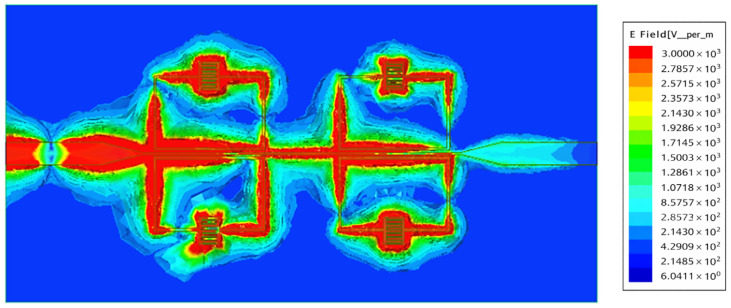
Magnitude distributions of the electric field on the top line coupled with the IDC-SRR at the resonant frequency.

**Figure 16:**
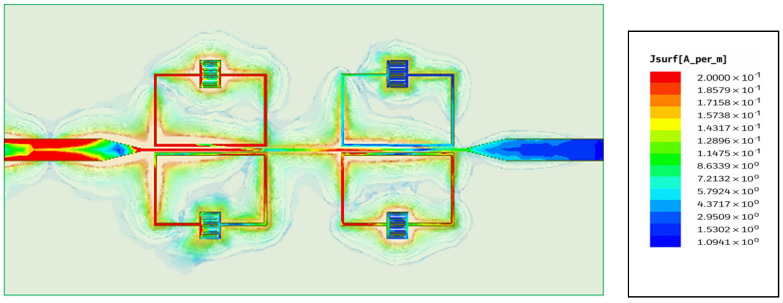
Simulated surface current distribution of the sensor at 3.46 GHz showing band-stop behavior.

The proposed sensor’s electric variations in the SRR-based IDC sensor at a particular frequency (3.42 GHz) are depicted in Figure 15. In the exact center of the edge that is farthest from the microstrip line, the resonance *LC* tank coupled to the microstrip line exhibits almost minimal electric field fluctuation, as shown in Figure 14. However, the IDC has the greatest electric field change, making it simple to examine the dielectric properties of MUT tissue. Figure 16 shows that the major role in determining the resonance behavior is confirmed by the current distribution plot at the resonant frequency of 3.46 GHz, which shows a strong concentration of surface current around the LC tank region. The structure efficiently supports resonance and stores the most electromagnetic energy in this region, as seen by the localized high current. On the other hand, the current intensity is much lower close to the second port, suggesting that little power is transferred outside of the resonator. This indicates that at this frequency, the structure functions as a band-stop filter. The sensor’s ability to reject signals outside of the desired resonance condition is supported by field confinement and current suppression.

### 3.3. Quantitative Analysis

**Figure 17:**
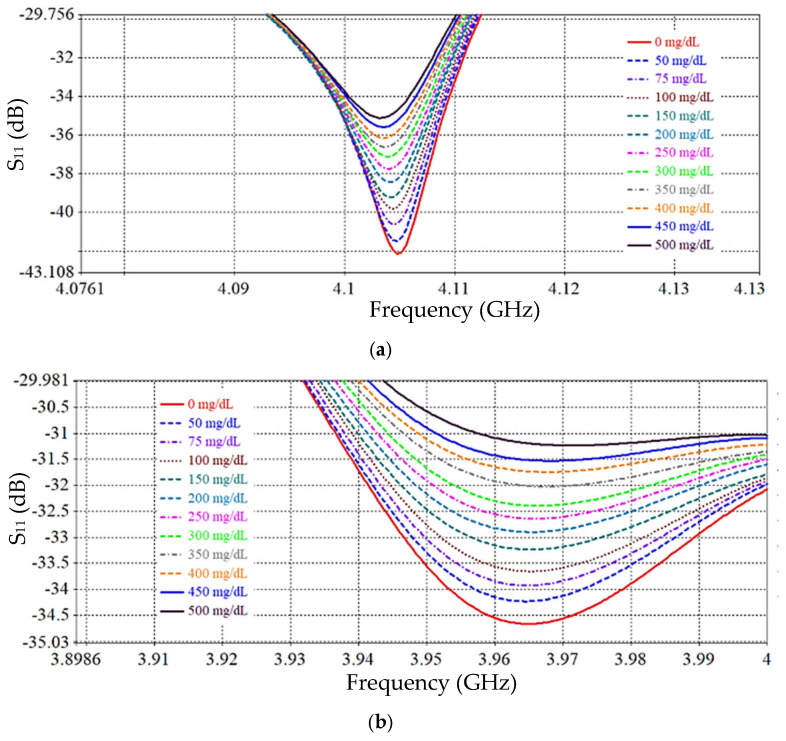
Reflection coefficient magnitude ∣S_11_∣ versus frequency for different glucose concentrations at (**a**) 4.1 GHz and (**b**) 3.9 GHz.

**Figure 18:**
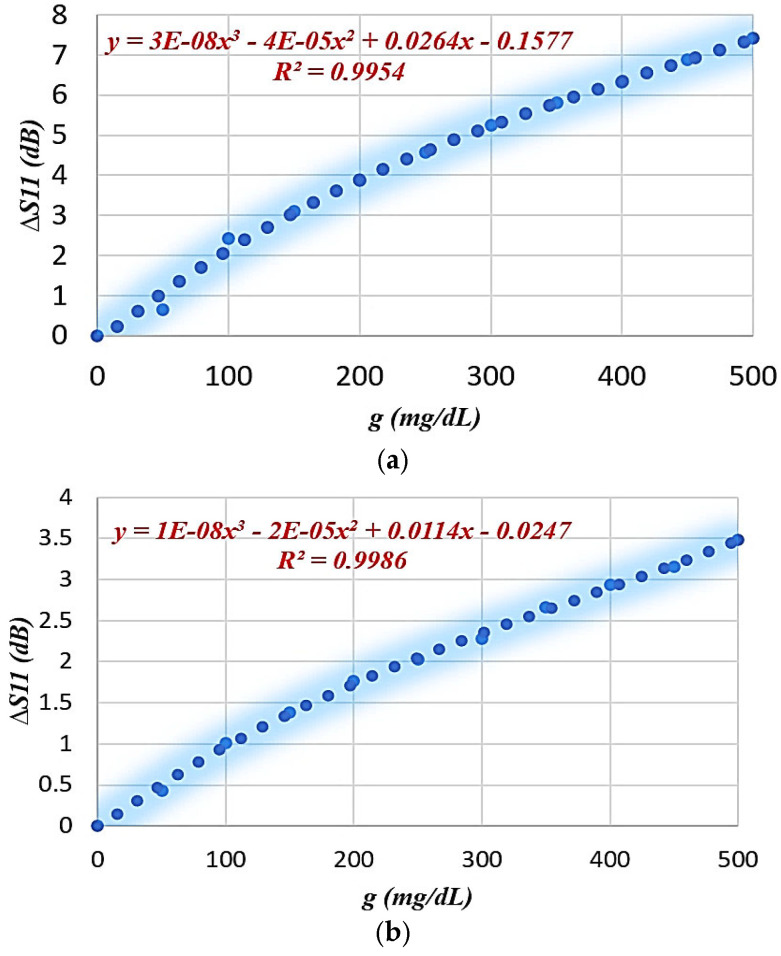
∆S_11_ variation with glucose constructions: (**a**) f = 4.1 GHz, (**b**) f = 3.9 GHz.

**Figure 19:**
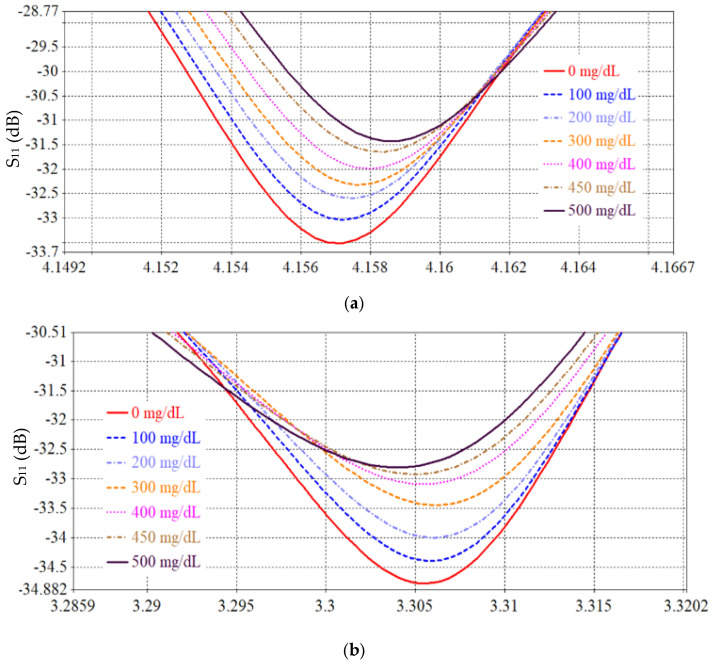
Transmission coefficient magnitude ∣S_21_∣ versus frequency for different glucose concentrations at (**a**) 4.1 GHz and (**b**) 3.9 GHz.

**Figure 20:**
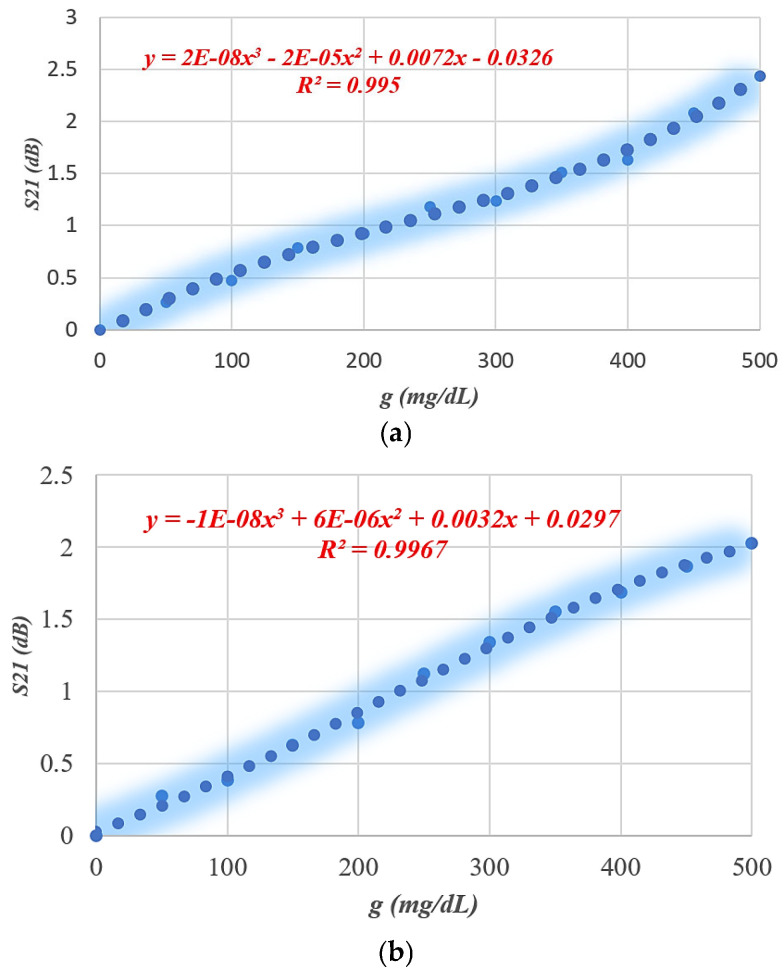
∆S_21_ variation with glucose constructions: (**a**) f = 4.157 GHz, (**b**) f = 3.305 GHz.

The performance of the proposed SRR-based IDC sensor is numerically analyzed based on the Finite Integration Technique (FIT) using computer simulation technology (CST 2019) software to evaluate its ability to monitor blood glucose levels non-invasively. A mock-up finger (MUT) was placed 0.5 mm above the *LC* tank surface to simulate realistic sensing conditions. To evaluate the effect of glucose concentration variations on the electromagnetic response, a parametric scanning analysis was performed covering eleven different concentrations from 0 to 500 mg/dL. Figure 17 and Figure 18 show the resulting S_11_ frequency response for different glucose concentration levels, where a clear change in the resonance depth and frequency is observed. Figure 19 and Figure 20 show the transmission (|S_21_|) response and the variation in the transmission ∆S_21_ magnitude with the glucose variation. Table 5 presents the average sensitivity of the proposed SRR-based IDC sensor at multiple resonance frequencies, evaluated using both reflection (S_11_) and transmission (S_21_) responses. In the reflection mode, the sensor exhibits its highest sensitivity of 0.015 dB/(mg/dL) at 4.1 GHz, while a lower sensitivity of 0.0071 dB/(mg/dL) is observed at 3.9 GHz. In comparison, the transmission mode demonstrates reduced sensitivity levels, with values of 0.005 dB/(mg/dL) at 4.157 GHz and 0.004 dB/(mg/dL) at 3.305 GHz. These results clearly indicate that the reflection-based sensing mechanism provides superior sensitivity compared to the transmission-based approach, owing to stronger electromagnetic field confinement within the sensing region introduced by the integrated interdigital capacitor (IDC) structure.

## 4. SRR-Based Moore Fractal Geometry Sensor

SRRs are used to design precise electromagnetic sensors that respond to subtle environmental changes, such as a change in the dielectric constant ε of biomaterials, which affects the resonance. Despite the success of conventional SRRs, improving sensitivity accuracy remains an ongoing challenge. To overcome this limitation, we propose Moore fractal geometry as a geometric modification of the SRR structure. This modification involves developing the loop through repeated, closed curves that enhance electromagnetic interaction with the surrounding bio-environment. In fractal geometry, the effective electrical length $l_{eff}=l_{o}N_{s}$, where $l_{o}$ is the original loop length and $N_{s}$ is the number of parts or segments in the fractal order. Using fractal geometry will enhance sensitivity to glucose changes. Slit variations in glucose concentration affect the dielectric constant ε of biological tissues, leading to a change in clearance capacity C. A more complex Moore structure leads to a larger interaction and, therefore, a higher sensitivity. The SRR has the ability to achieve multiple resonance frequencies within the same physical footprint. This is because fractal geometries inherently introduce multiple spatial scales into the resonator’s structure, which results in the excitation of several electromagnetic modes. Unlike conventional SRRs, which typically resonate at a single frequency determined by their effective inductance (*L_eff_*) and capacitance (*C_eff_*), fractal-based SRRs—especially those employing recursive and space-filling geometries like the Moore curve—exhibit a cascade of resonance modes corresponding to each fractal iteration. This enables the sensor to operate at several frequencies simultaneously, improving its spectral sensitivity and quality factor and allowing for more complex sensing capabilities. There is a fundamental resonance condition for each mode. When this type of SRR-MORE is placed on human skin, over areas rich in capillaries (such as the arm or wrist), changes in the resonance frequency resulting from subtle changes in the permittivity coefficient associated with the glucose concentration in the interstitial fluid can be tracked. Using a low-power transmitter/receiver or a small beamforming network analyzer, the resonant frequency can be precisely determined, enabling non-invasive glucose calculations without the need for blood draws.

### 4.1. Moore Fractal Curve Generation

The Moore curve is a continuous space-filling fractal curve (SFC) and can be considered a looped variant of the classical Hilbert curve. While the Hilbert curve starts at a corner of the unit square (e.g., (0, 0)), the Moore curve begins from the midpoint of the bottom edge of the square, specifically at the point (1/2, 0), and it ends at the same point, forming a closed loop [39]. Let I = [0, 1] ⊂ ℝ be the unit interval and I^2^ = [0, 1] × [0, 1] ⊂ ℝ^2^ be the unit square. The Moore curve is recursively defined as a sequence of polygonal approximations Mn: I → I^2^, where n ∈ ℕ_0_.

Base cases:

- For n = 0: M_0_: = H_0_ = (1/2, ½);
- For n = 1: M_1_: = H_1_, where H_1_ is the first-order Hilbert curve.

Recursive step:

For all n ≥ 2, the Moore curve Mn is constructed using:

- Two copies of the Hilbert curve H_n−1_ rotated counterclockwise by 90°, denoted θ(H_n−1_);
- Two copies of H_n−1_ rotated clockwise by 90°, denoted θ^−1^(H_n−1_);
- All scaled by a factor h_n_ = (1/2)^n^ and arranged in a clockwise order;
- Connected by three additional line segments of length h_n_.

This recursive assembly ensures that Mn forms a closed loop for every n ≥ 2, converging to the continuous Moore curve M = lim_n_ → ∞ Mn.

Given two continuous paths γ_1_, γ_2_: [0, 1] → ℝ^n^, such that γ_1_ (1) = γ_2_ (0), the concatenated path γ_1_ ∗ γ_2_ is defined by:

(10) $$\left(\gamma_{1}*\gamma_{2}\right)(t)=\left\{\begin{array}{c} \gamma_{1}\left(2t\right)\;\;\;\;\;\;\;\;\;\;\;\;\;\;\;for\;0\leq t\leq \frac{1}{2}\\ \gamma_{2}\left(2t-1\right)\;\;\;\;\;\;for\;\;\frac{1}{2}\leq t\leq 1 \end{array}\right.$$

The number of line segments in the Hilbert curve at iteration n is:

(11) $$L_{H}\left(n\right)=2^{n-1}$$

For the Moore curve, the closed-loop structure increases the number of segments by one:

(12) $$L_{M}\left(n\right)=2^{n}$$

Each segment has a length of:

(13) $$l_{n}\left(n\right)={\left(\frac{1}{2}\right)}^{n}$$

(14) $${length}_{M}\left(n\right)=L_{M}\left(n\right)\cdot l_{n}=2^{n}$$

### 4.2. Developing Moore SRR Loop

**Figure 21:**
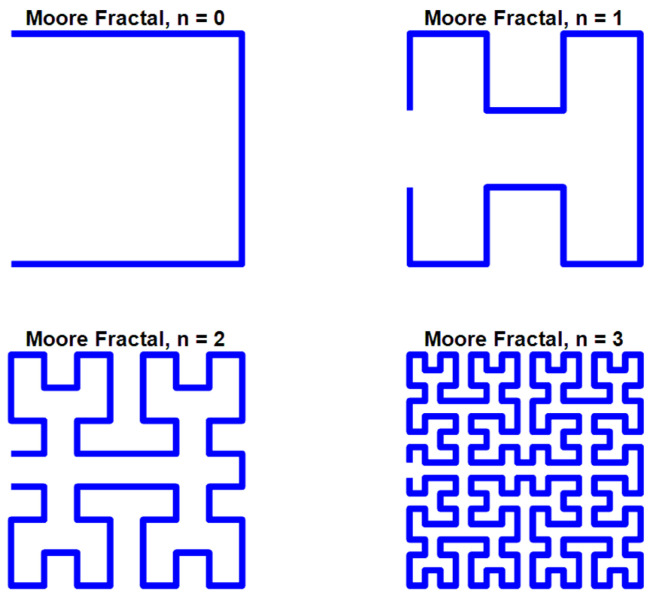
Geometric evolution of the Moore fractal curve through four consecutive iterations (n = 0–3).

**Figure 22:**
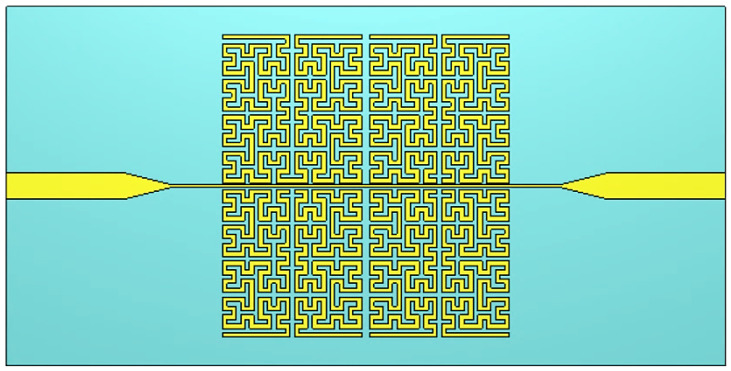
SRR-based Moore fractal curve sensor geometry.

Figure 21 illustrates the geometric evolution of the Moore fractal curve through four successive iterations (n = 0–3), highlighting the increasing structural complexity and effective electrical length. Figure 22 shows the final design of an SRR loop that uses Moore fractal geometry. The design began with a simple U-shape, which was the first iteration (Iteration 1) in the void structure. Then, a fractional transformation was applied to the fourth iteration (Iteration 4) based on the Moore curve. This curve has the property of filling the space more densely while keeping the same geometric dimensions. The SRR design used fractal geometry to make the current path longer in a small space. This technique lowers the resonant frequency without having to make the resonator bigger. This makes it easier to design compact systems and gives a more accurate frequency response when working with different biological or environmental materials. Using a Mohr curve, in particular, makes branching more even and repetitive. This makes the electromagnetic field lines in the cavity denser and increases electromagnetic coupling. The design was made using FR-4 as the substrate. It was 1.6 mm thick and had a relative dielectric constant εr of 4.4 and a loss tangent of about 0.02. To make sure that the two designs could be compared accurately, the remaining geometric dimensions of the cracks and traces were kept the same as those of the standard SRR reference design. This engineering change should greatly improve the resonator’s sensitivity and Q-factor, as well as its ability to create more than one resonant frequency in the same range. This makes the design suitable for multi-frequency sensing applications.

**Figure 23:**
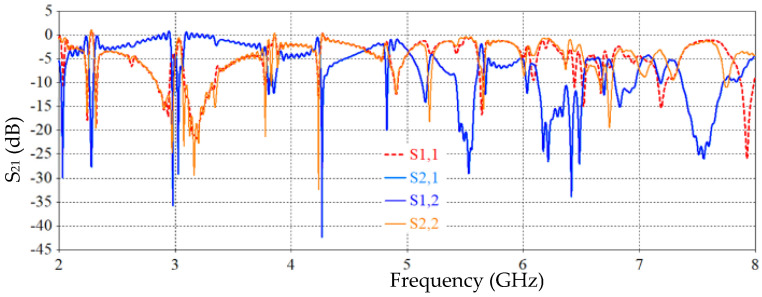
S-parameter responses of the proposed SRR-based Moore fractal curve sensor.

The results from Figure 23 show that the Moore fractal SRR-based sensor’s propagation parameter response (S-parameters) is more complex and detailed than that of the standard design. We can see a clear rise in the number of resonant dips, and the depth of these dips is sharper, especially in the coefficients S_21_ and S_12_. This means that electromagnetic coupling and response sensitivity have improved. The fractal design was able to create more resonances in the same frequency range (2–8 GHz) than the traditional SRR design. This makes it more useful for multi-frequency sensing applications. The sharper dips also show that the quality factor (Q-factor) has improved, which is important for making sensing more accurate. This improvement shows how effective fractal geometry is at making electromagnetic sensors work better without changing the size of the resonator.

**Figure 24:**
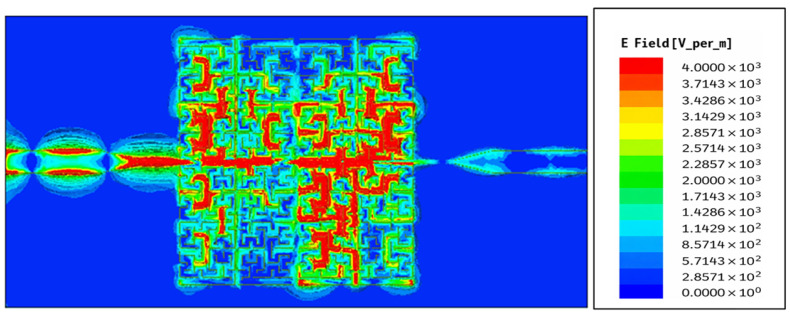
Magnitude distributions of the electric field on the top line coupled with the SRR-based Moore fractal curve at the resonant 4.27 GHz frequency.

Figure 24 shows how the electric field E of the proposed fractal resonator is spread out at a frequency of 4.27 GHz. The electric field is very strong in the areas of the micro-slits and zigzags created by the fractal Moore geometry, especially at the edges of the curve and along its complicated paths. The dense field distribution (in red) shows that there are areas where a lot of electromagnetic energy is stored, which is why the response is so strong at this frequency. This behavior is a common trait of fractal geometries, which allows electromagnetic interference to work better in a small area.

**Figure 25:**
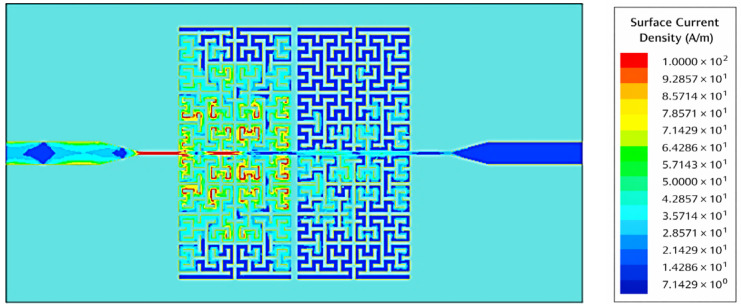
Simulated surface current distribution of the sensor at 4.26 GHz showing band-stop behavior.

Figure 25 shows the distribution of the surface current density $\overset{⃑}{J_{surf}}$ at the conductor surface at the same frequency. We see that the surface currents are spread out unevenly and strongly in zigzag patterns, with a lot of them in the left part of the resonator. This shows that the resonator absorbs a lot of electromagnetic energy and stops it from passing through the sensor. This distribution corresponds to a sharp drop in the transmission coefficient *S*_21_, S_21_, which reaches −42 dB. This means that the resonator works as a band-stop filter at this frequency, effectively blocking the signal.

Thus, the introduction of the fractional Moore curve geometry not only achieves multiple resonant frequencies but also improves energy localization and current concentration in effective regions, a significant improvement in the performance of the electromagnetic sensor.

### 4.3. Quantitative Evaluation for the SRR-Based IDC Sensor

**Figure 26:**
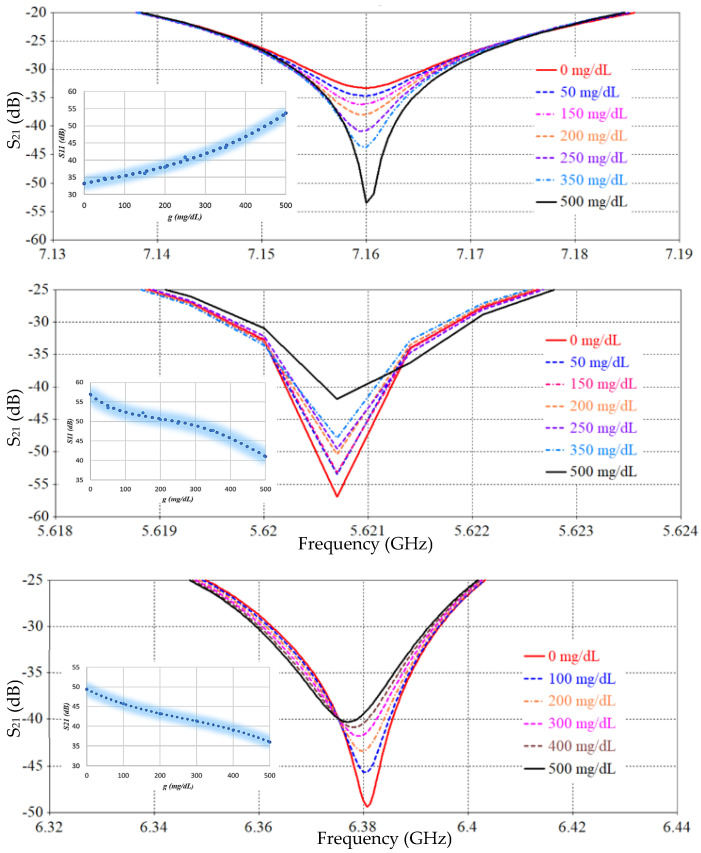
S_11_ response for different glucose levels.

**Figure 27:**
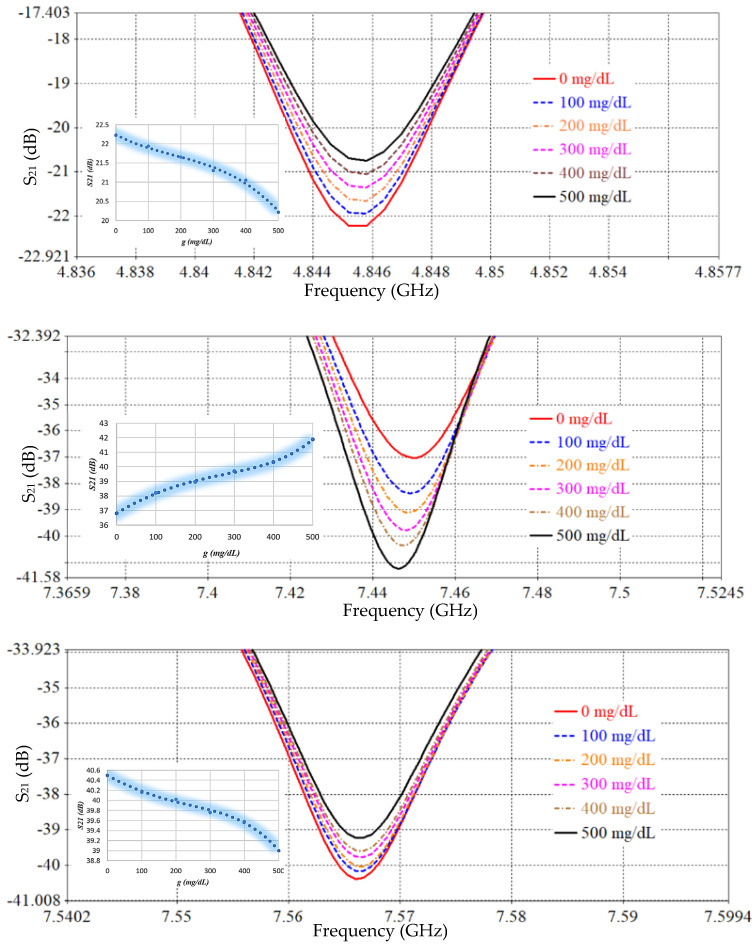
Simulated reflection coefficient (S_11_) as a function of frequency for various glucose concentrations, illustrating resonance shift and magnitude variation at distinct operating bands. Insets depict the extracted sensitivity behavior.

The performance of the proposed SRR-based Moore fractal curve sensor is numerically analyzed based on the Finite Integration Technique (FIT) using computer simulation technology (CST) software to evaluate its ability to monitor blood glucose levels non-invasively. A mock-up finger (MUT) was placed 0.5 mm above the *LC* tank surface to simulate realistic sensing conditions. To evaluate the effect of glucose concentration variations on the electromagnetic response, a parametric scanning analysis was performed covering eleven different concentrations from 0 to 500 mg/dL. Figure 26 and Figure 27 show S_11_ and S_21_ spectra, respectively, for different glucose concentration levels. We note that there are six resonance frequencies affected by changes in glucose, and this sets the SRR sensor based on the Moore curve of fractal geometry apart from earlier models.

Table 3, Table 5 and Table 6 present a comprehensive comparison of the sensor’s sensitivity and frequency changes at S_11_ and S_21_ spectra for different SRR resonator designs. Table 3 represents the conventional SRR design, Table 5 represents a partially improved design based on IDC, and Table 6 reflects the proposed design based on the fractional Moore curve geometry. The conventional SRR design (Table 3) exhibits a very low sensitivity, not exceeding 0.0052 dB/(mg/dL) at the best possible time, at a resonant frequency of 1.7 GHz. The design in Table 5 has improved performance, reaching 0.015 dB/(mg/dL) at 4.1 GHz, with an increased number of effective resonant frequencies. In contrast, the design based on the fractal Moore curve geometry shows a qualitative and quantitative improvement in sensor performance. The highest sensitivity was recorded at 7.16 GHz in the obtained S_11_ spectrum, representing an improvement of approximately eightfold compared to the conventional design. A large diversity of resonance frequency locations was also observed, and the sensitivity was distributed across multiple resonance modes, enhancing the sensor’s effectiveness in multi-frequency detection and increasing its accuracy in biological applications. This significant performance improvement is attributed to the increased effective path for electromagnetic current in the fractal design, which increases interference efficiency and response when exposed to changes in the surrounding medium, such as glucose concentration. Therefore, the fractal design provides a promising platform for improving small-scale, high-efficiency resonant sensors.

Table 6 summarizes the average sensitivity of the proposed SRR-based Moore fractal geometry sensor across multiple resonance frequencies in both reflection (S_11_) and transmission (S_21_) modes. A clear enhancement in sensing performance is observed compared to previous designs, with the highest sensitivity of 0.042 dB/(mg/dL) achieved at 7.16 GHz in the reflection mode. Additional S_11_ resonances at 5.62 GHz and 7.446 GHz exhibit sensitivities of 0.032 and 0.0102 dB/(mg/dL), respectively, demonstrating the multi-resonant nature of the Moore fractal structure. In the transmission mode, the sensor shows multiple resonances, with sensitivities reaching up to 0.03 dB/(mg/dL) at 6.389 GHz. The overall results confirm that the Moore fractal geometry significantly enhances electromagnetic field localization and effective current path length, leading to superior sensitivity and enabling reliable multi-frequency glucose sensing within a compact footprint. A comparative analysis of the proposed antenna with previously reported works is presented in Table 7.

## 5. Benchmarking Sensor Sensitivity Against Existing Microwave-Based Designs

**Figure 28:**
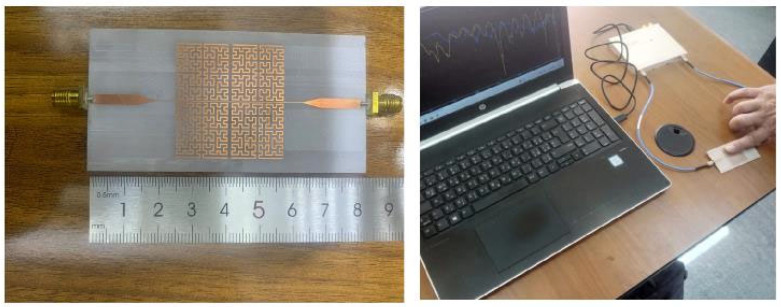
Fabricated fractal-based microwave sensor and experimental measurement setup for non-invasive glucose testing.

**Figure 29:**
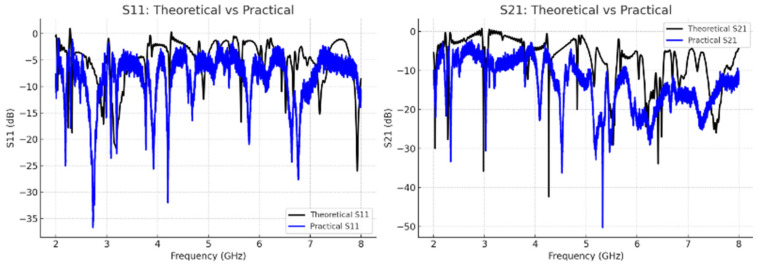
Comparison of simulated and measured S-parameters (S_11_ and S_21_).

A wide range of previously reported microwave-based sensors in terms of operating frequency and sensitivity. As summarized in Table 6, most of the existing designs offer sensitivity values ranging between 0.000003 and 0.027 dB/(mg/dL), with only a few sensors achieving sensitivities above 0.005. Notably, the proposed SRR-based Moore fractal curve sensor demonstrates a remarkable sensitivity of 0.042 dB/(mg/dL), which significantly outperforms the vast majority of reported sensors. This is especially impressive considering that many high-sensitivity sensors operate at millimeter-wave frequencies (e.g., [44] at 60 GHz), whereas our design achieves its performance in the more practical microwave band (~4–7.5 GHz). Furthermore, even when compared to other SRR-based designs, such as [33,52], our sensor exhibits a substantial enhancement in sensitivity, indicating that the integration of the Moore fractal geometry with SRR architecture leads to a more effective interaction with the target analyte. These results underscore the potential of the proposed design for highly sensitive and compact biosensing applications. Figure 28 illustrates the fabricated fractal sensor prototype together with the measurement setup, where the sensor is connected to a vector network analyzer (VNA) and tested using fingertip placement on the resonator surface. Figure 29 presents the comparison between simulated and measured S-parameters (S_11_ and S_21_). The practical results exhibit a good agreement with simulations, with slight discrepancies attributed to fabrication tolerances and measurement noise. This consistency validates the reliability of the proposed sensor design.

Accordingly, the claims related to clinical applicability have been carefully moderated, and the proposed sensor is positioned as a proof of concept demonstrating strong potential for future non-invasive glucose monitoring rather than immediate clinical deployment. A dedicated discussion has been added to emphasize that further experimental and clinical investigations are required before real-world translation.

In addition, a brief discussion has been incorporated addressing practical factors that may influence on-body sensing performance, including tissue heterogeneity, motion-induced artifacts, and temperature variations. While these effects were not explicitly modeled in the present numerical framework, they are recognized as critical considerations for future experimental validation and system-level optimization. The inclusion of this discussion strengthens the realism of the proposed approach and outlines clear directions for subsequent work.

In the present work, a small separation distance of approximately 1 mm between the fingertip and the sensor surface is intentionally assumed to minimize excessive electromagnetic field distortion at the skin interface. This design consideration is particularly important in on-body microwave sensing applications, where direct contact with the electrically resistive epidermis can significantly degrade field distribution and sensing accuracy. Recent studies have confirmed that uncontrolled skin contact leads to substantial electromagnetic field distortion and reduced penetration depth, adversely affecting sensor performance. In this context, Ref. [57] demonstrated that mitigating field distortion either through geometrical modification or controlled separation plays a critical role in enhancing on-body sensing reliability. Building on this insight, the proposed Moore fractal SRR-based sensor leverages enhanced electromagnetic field confinement and multi-resonant behavior to achieve high sensitivity while maintaining a stable and realistic sensor-to-skin spacing, thereby ensuring reliable non-invasive glucose sensing without requiring direct skin contact.

## 6. Conclusions

This study showed how to build an ultra-sensitive microwave sensor that can measure blood glucose levels without having to touch the skin, using a fractal-shaped SRR structure. In the first phase, a straightforward, standard SRR design was utilized, demonstrating a singular resonant frequency in the transmission mode at 2.975 GHz, accompanied by a low sensitivity of 0.0032 dB/(mg/dL), inadequate for medical applications necessitating high detection precision. In the second phase, an IDC was added to the resonant structure to further improve the design. This helped to make the electromagnetic field inside the sensor stronger. This led to a big improvement in performance, with a clearer resonant frequency at 4.1 GHz and a higher sensitivity of 0.015 dB/(mg/dL), showing that the system was starting to respond well to changes in glucose concentration. The third stage saw a huge improvement in performance because the SRR design used fourth-order Mohr fractal geometry. This led to six resonant frequencies in the high band, from 4.84 to 7.56 GHz. A resonant frequency was measured at 4.84 GHz with a sensitivity of 0.012 dB/(mg/dL), and another was measured at 5.75 GHz with a sensitivity of 0.017 dB/(mg/dL). A frequency at 6.37 GHz with a sensitivity of 0.021 dB/(mg/dL) came up next, and then a frequency at 6.84 GHz with a sensitivity of 0.028 dB/(mg/dL). The sensitivity reached its highest point at 7.16 GHz, when it reached 0.042 dB/(mg/dL), the highest value found in the experiments. Last but not least, a frequency of 7.56 GHz had a sensitivity of 0.031 dB/(mg/dL). The fractal structure’s ability to increase electromagnetic interaction and make the sensor more effective while keeping its size small and stable (80 mm × 40 mm × 1.6 mm) is shown by the significant rise in the number of resonant frequencies and their response to changes in the bio-measurement medium. The proposed design effectively detects subtle changes in glucose concentration without requiring any invasive intervention. This makes it a great choice for use in smart and autonomous medical monitoring systems. So, we can say that using advanced fractal geometries to design the SRR resonator is a promising way to make biosensors that are very sensitive and very accurate for use in modern healthcare, especially for monitoring blood glucose levels without having to cut into the skin.

## Figures and Tables

**Table 1:** Proposed SRR sensor dimensions.

Cell Parameter	*L_s_*	*W_s_*	*L*	*w*	*w_f_*	*L_f_*	g	$g_{1}$	*h*
Description	Substrate length	Substrate width	Ring trace length	Ring trace width	Feedline width	Feedline length	Stub gap	SRR split gap	Substrate thickness
Dimension (mm)	80	40	10	4.9	2.9	13	1	0.3	1.6

**Table 2:** Lumped elements’ extracted parameters.

Lumped Elements’ Value	*L_L_* (nH)	*C_L_* (PF)	*L_r_* (nH)	*C_r_* (PF)
Dimension (mm)	3	1.14	1.8	2

**Table 3:** Sensor average sensitivity.

Sensor Parameter	*S*_21_	*S*_11_
Resonance frequency (GHz)	2.975	1.7
Average sensitivity dB/(mg/dL)	0.0032	0.0052

**Table 4:** Proposed SRR-based IDC sensor dimensions.

Structure Parameter	*L_s_*	*W_s_*	*l*	*w*	*L_fing_*	*w_fig_*	*w_f_*	*L_f_*	g	$g_{1}$	*h*
Dimension (mm)	80	40	14.7	10	2	0.2	2.9	13	1	0.3	1.6

**Table 5:** Sensor average sensitivity.

Sensor Parameter	*S*_11_	*S*_11_	*S*_21_	*S*_21_
Resonance frequency (GHz)	4.1	3.9	4.157	3.305
Average sensitivity dB/(mg/dL)	0.015	0.0071	0.005	0.004

**Table 6:** SRR-based Moore fractal geometry sensor average sensitivity.

Parameter	*S*_11_	*S*_11_	*S*_11_	*S*_21_	*S*_21_	*S*_21_
Frequency (GHz)	7.16	5.62	7.446	7.566	6.389	4.84
Sensitivity dB/(mg/dL)	0.042	0.032	0.0102	0.003	0.03	0.004

**Table 7:** Performance comparison of the proposed antenna with the literature.

Reference	Technology	Frequency (GHz)	Sensitivity dB/(mg/dL)
[40]	Flexible microstrip resonator	0.93	0.00194
[41]	CSRR resonator	2.95	0.000003
[42]	Open-ended microstrip transmission line loaded with CSRR	2.5	0.00005
[43]	Hilbert-shaped microwave sensor	6.1	0.0000156
[44]	Millimeter waves using microstrip patch antennas	60	0.65 ×10^−3^
[24]	Loaded patch resonator	2.45	0.0033
[45]	Corona-shaped resonator	1.935	0.0057
[42]	Microwave reflective biosensor	2.5	0.005
[46]	Metamaterial microwave sensor	4.3	0.027
[33]	Hyper-sensitive based on the SRR	1.85	0.00042
[47]	Coplanar waveguide resonator integrated with a microfluidic channel	1.9	0.00023
[48]	Parallel resonators	2.5	0.0005
[49]	Defective–ground–structure coplanar waveguide	2.2	0.005
[50]	Miniature microstrip line-based sensors	7.25	0.0062
[51]	Microwave-based microfluidic sensor	7.5	7.6 × 10^−5^
[52]	Coplanar waveguide transmission line with electric-LC resonator	3.41	0.03125
[53]	Microstrip line-based	1.48	(1.8–6.6) × 10^−3^
[54]	Linear and mediator-free resonator	1.5	0.0049
[55]	Deep learning enhanced wearable microwave sensor	7.8	0.015 dB/mg/dL
[56]	Modified inductive stub-coupled CSRR	2.3	0.086 MHz/mg/dL 0.02 dB/mg/dL
[57]	Dual-band CSRR	2.45 and 5.8	0.0075
This Work	Congenital SRR sensor	~4–7.5	0.0052
	SRR-based IDC sensor		0.015
	SRR-based Moore fractal curve sensor		0.042

## Data Availability

The original contributions presented in this study are included in the article. Further inquiries can be directed to the corresponding author.
